# Artificial intelligence applications in intracerebral hemorrhage care: implications for clinical and nursing practice - a narrative literature review

**DOI:** 10.3389/fresc.2025.1620335

**Published:** 2025-07-07

**Authors:** Seoyoung Kim, Jungmin Lee, Soo-Hyun Nam

**Affiliations:** ^1^ Department of Artificial Intelligence Convergence, Graduate School, Hallym University, Chuncheon, Republic of Korea; ^2^ School of Nursing, Hallym University, Chuncheon, Republic of Korea; ^3^ School of Nursing Science, Gyeongkuk National University, Andong, Republic of Korea

**Keywords:** artificial intelligence (AI), intracerebral hemorrhage (ICH), machine learning, prognosis prediction, functional outcome, rehabilitation

## Abstract

Little is known about how artificial intelligence tools are utilized across the different stages of intracerebral hemorrhage care or how they contribute to clinical decision-making and patient outcomes in this population. This narrative review aimed to explore current applications of artificial intelligence in the clinical management of patients with intracerebral hemorrhage. A comprehensive search was conducted across five electronic databases (PubMed, CINAHL Plus with Full Text, Ovid MEDLINE, ProQuest, and Web of Science), supplemented by additional manual searches. This review included studies published in English between January 1, 2014, and December 31, 2024. Seven studies examining the application of artificial intelligence in the acute and post-acute phases of intracerebral hemorrhage care were included. In the acute phase, machine learning models such as Random Forest and XGBoost outperform traditional prognostic scoring systems, offering clinicians more precise tools for early risk stratification. In the post-acute phase, AI contributes to continuity of care by supporting data completion, rehabilitation planning, and remote rehabilitation, thereby enhancing patient-centered nursing practice with high predictive accuracy and practical utility. These findings suggest that artificial intelligence holds significant promise for enhancing prognosis prediction, clinical decision-making, and continuity of care in patients with intracerebral hemorrhage.

## Introduction

The rapid advancement of digital technologies is transforming healthcare delivery, particularly through the integration of artificial intelligence (AI) into clinical practice (1). AI encompasses a range of tools, including machine learning algorithms, big data analytics, radiomics, telehealth platforms, and wearable devices, which have demonstrated the potential for enhancing clinical decision-making, operational efficiency, and patient outcomes (2, 3). These innovations are being increasingly explored for the management of acute neurological conditions, including intracerebral hemorrhage (ICH) (4).

ICH is a severe and life-threatening form of stroke, characterized by spontaneous bleeding within the brain parenchyma (5). Due to its high morbidity and mortality rates, the management of ICH demands rapid diagnosis, continuous monitoring, and individualized care strategies across both the acute and post-acute phases (6). The complexity of ICH care presents challenges in early prognosis, treatment planning, and rehabilitation support (7). AI technologies offer innovative solutions in these areas through real-time monitoring, predictive modeling, and automated decision support systems (8).

Although numerous studies have examined AI applications in stroke care (9), most have focused on ischemic stroke or general stroke populations (10), with limited attention paid to ICH-specific interventions. In addition, little is known about how AI tools have been utilized across different stages of ICH care (e.g., at admission vs. during rehabilitation) or how they contribute to clinical decision-making and patient outcomes in this population (11, 12).

Therefore, this narrative review aimed to synthesize the current evidence on AI applications in the clinical care of patients with ICH. The objectives were to (1) identify the clinical contexts and care stages in which AI technologies have been applied to ICH, (2) describe the types of AI methods used, and (3) summarize the reported outcomes of these interventions. Although this review initially aimed to include nursing-specific applications, the final selection of studies did not directly address nursing practice. Nonetheless, these findings offer insights relevant to interdisciplinary teams, including implications for future nursing integration into AI-supported neurological care.

## Methods

### Design

This study was conducted as a narrative literature review to explore the current applications of AI in the clinical management of patients with ICH. A narrative approach was selected owing to the interdisciplinary and evolving nature of AI in healthcare, which spans diverse methodologies, study designs, and application contexts. Unlike systematic reviews that aim to synthesize high-level quantitative data, this narrative review—guided by the methodological approach proposed by Baumeister and Leary (1997)—allows the integration of heterogeneous evidence and provides a structured overview of the phenomenon of interest (13). This approach also enabled the identification of conceptual trends, methodological gaps, and emerging areas for clinical implementation.

### Searching and selecting literature

A systematic literature search was conducted between January 2024 and December 2024 across five electronic databases: PubMed, CINAHL Plus with Full Text, Ovid MEDLINE, ProQuest, and Web of Science. The keywords and indexing terms were “intracerebral hemorrhage,” “nursing care,” “artificial intelligence,” “machine learning,” “telemedicine,” “wearable devices,” and “remote monitoring.” Additional terms such as “patient-centered care,” “nursing interventions,” and “digital health innovation” were also applied to capture a broad range of literature. Boolean operators (AND, OR) were used to combine search terms and optimize retrieval of relevant studies. The search strategy for the included databases is presented in Supplementary File 1.

Although the search strategy initially included terms related to nursing care, the selection of eligible studies was not limited to those that directly addressed nursing-specific interventions. To minimize the risk of missing relevant studies due to variability in terminology or database indexing, a supplementary manual search was conducted. This involved keyword-based browsing of relevant journals and backward reference tracing of key articles, without relying on formal Boolean queries.

### Inclusion and exclusion criteria

This review included studies published in English between January 1, 2014, and December 31, 2024, that examined the application of artificial intelligence in the clinical care of patients with ICH. Eligible studies focused on AI-based tools used at various stages of ICH management, including technologies such as machine-learning algorithms, clinical decision support systems, radiomics, wearable devices, telemedicine platforms, and remote monitoring.

The primary inclusion criteria were: (1) the application of AI to ICH-related clinical care and (2) the reporting of measurable outcomes, such as prediction accuracy, data quality, rehabilitation outcomes, or functional status. Studies were not required to focus exclusively on nursing interventions but were considered if the findings were relevant to clinical decision-making or patient management. The list of excluded full-text studies and the reasons for exclusion are presented in Supplementary File 2.

Studies were excluded if they (1) were not published in English; (2) were classified as editorials, opinion pieces, or letters to the editor; (3) focused exclusively on animal models or preclinical research; or (4) did not include the use of AI technologies in relation to ICH patient care.

**Figure 1:**
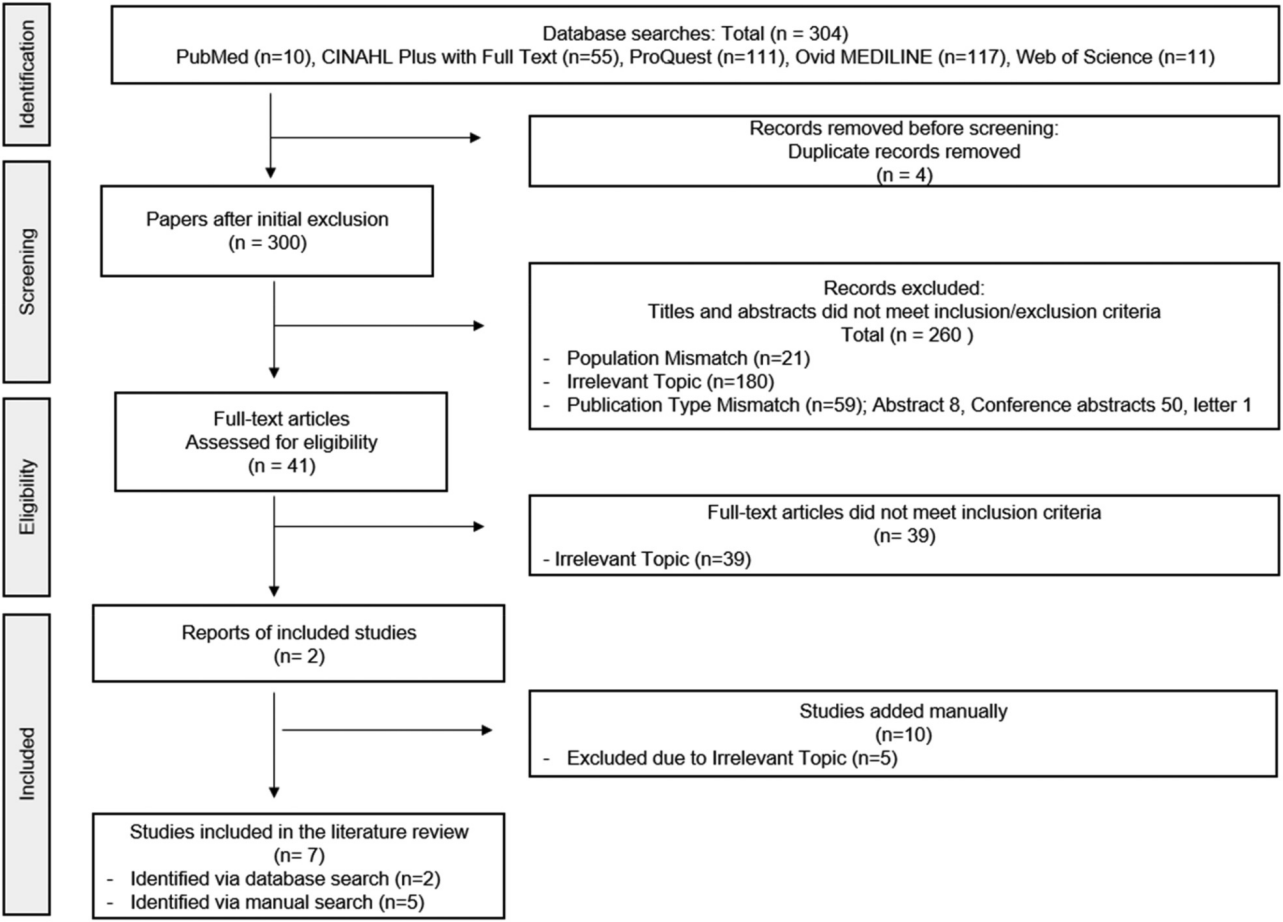
Study screening and selection process.

To ensure methodological rigor and eliminate redundancy, duplicate articles were removed using EndNote X20 (Clarivate Analytics, Philadelphia, USA). Study selection followed a structured process, starting with title and abstract screening for relevance. A full-text review of eligible studies was conducted. Two independent reviewers assessed each article, and disagreements were resolved through discussion or consultation with a third reviewer to ensure objectivity. The overall study screening and selection process is illustrated in Figure 1.

### Data extraction

The initial data extraction was independently performed by the first author, and a secondary review was carried out by another co-author to ensure accuracy. Data from each included study were collected using a standardized extraction form. When further details were required, the corresponding authors were contacted via email.

## Results

### Study selection

In total, 304 articles were identified. After the removal of four duplicates, 300 records underwent title and abstract screening. Of these, 260 were excluded due to population mismatch, irrelevance to the topic, or publication type. The remaining 40 full-text articles were reviewed, and 38 were excluded based on the inclusion criteria. Two studies were included from the database search. This stringent selection was necessary to ensure that only studies with direct clinical applicability of AI in ICH and well-defined outcome measures were retained, thereby enhancing the validity and relevance of the findings. Common reasons for exclusion included lack of clinical application of AI, insufficient outcome reporting, and studies limited to theoretical modeling without validation. We also acknowledge that the number of studies combining ICH and nursing-specific AI interventions is limited in the current literature. In many cases, ICH is classified within broader stroke-related research, and relevant AI applications are embedded under generalized cerebrovascular care frameworks, which constrained the final inclusion count despite a comprehensive search strategy. To mitigate the risk of overlooking relevant studies, two reviewers independently conducted the screening process, and discrepancies were resolved through discussion with a third reviewer. This procedure aimed to enhance transparency and consistency in the application of the inclusion criteria.

A manual search identified 10 additional studies, of which five met the eligibility criteria. Combined with two studies identified through database searches, a total of seven studies were included in the final literature review (Figure 1 and Table 1).

**Table 1:** Key characteristics of the included studies.

Authors; Location	Purpose	Design; Data collection	ICH phases	Applied AI	Results	Key findings
Katsuki et al. (6); Japan	Predict 6-month functional outcome after ICH surgery using DL	Retrospective study; *N* = 140 (100 training, 40 validation); post-operative patients with hypertension and ICH	Post-surgical phase; predict 6-month functional outcome	Deep learning (Prediction One, Sony; proprietary, auto-optimized model; architecture not publicly disclosed)	AUC = 0.997 (train), 0.884 (validation); Accuracy = 80%	DL outperformed ICH, FUNC, and ICH grading scores; CRP, ALT, eosinophils identified as strong predictors
Nie et al. (14); China	Predict in-hospital mortality using ML	Retrospective study; *N* = 760 patients with ICH from MIMIC-III; no control group; ICU data within 24 h	Acute phase; predict in-hospital mortality using ICU admission data	Machine learning (Random Forest, AdaBoost, etc.) on early physiological/clinical data	RF AUC = 0.819; APACHE II AUC = 0.423	RF outperformed APACHE II; early ICU data as strong predictors
Sonobe et al. (15); Japan	Predict post-rehab functional outcomes	Retrospective cohort; *N* = 100 patients with ICH; post-rehabilitation; no control group	Post-acute phase; predict discharge-level functional outcome after inpatient rehabilitation	Balanced Random Forest using clinical and imaging variables	AUCs: 0.952 (good), 0.790 (moderate), 0.921 (poor)	Ternary outcome classification using post-rehab data is rare and detailed
Wang et al. (16); China	Evaluate ML imputation on discharge data	Simulation study; *N* = 1468 ICH cases; discharge data with missing value scenarios; no control group	Post-acute phase; evaluate ML-based imputation on discharge assessment for ICH patients	Ensemble Learning vs. KNN, MICE, Logistic Regression (missing data simulation)	AUC = 0.924; Sensitivity = 0.908; Kappa = 0.596	Focused on data quality via imputation; addressed missing data rigorously
Xu et al. (17); China	Predict 6-month prognosis using CT radiomics	Retrospective study; *N* = 270 HICH patients; CT radiomics + clinical data; no control group	Acute phase; predict 6-month prognosis using radiomics features at admission	CT radiomics + Random Forest, XGBoost, logistic regression	RF: Sensitivity = 93.3%, Specificity = 92.5%, Accuracy = 92.7%	Used high-dimensional radiomics features; very high model performance
Zhang et al. (18); China	Evaluate telerehab effect on exercise adherence	Multicenter RCT protocol; planned *N* = 332 patients with hemorrhagic stroke; ITECP vs. routine rehab	Post-acute phase; evaluate ITECP effect on exercise adherence post-discharge	Mobile-based ITECP platform via WeChat for remote rehab	RCT ongoing; expected outcomes include improved adherence, strength, QoL; results pending	Only RCT protocol; focused on telerehabilitation in younger population
Zhou et al. (19); China	Develop radiomics-clinical nomogram for 30-day prognosis	Retrospective study; *N* = 326 patients with deep ICH; CT radiomics + clinical data; model validation with internal/external cohorts	Acute phase; predict 30-day prognosis using admission CT and clinical data	Radiomics + clinical features; nomogram via logistic regression	AUCs: 0.80 (development), 0.79 (test), 0.70 (validation)	Combines radiomics with clinical variables; model externally validated

### Characteristics of included studies

A predefined format table was developed to summarize the key characteristics of the included studies, including the research design, study objectives, data sources, AI methods, sample size, and quantitative outcomes (Table 1). Each article was fully reviewed to ensure an accurate understanding of its content and relevance to the review objectives.

Data were extracted using Microsoft Excel. The studies were categorized by the stage of ICH care in which AI was applied (pre-ICH or post-ICH) and by the type of AI methodology utilized (e.g., radiomics, imputation models, telerehabilitation). This categorization provides a structured framework for comparing clinical contexts and reported outcomes across studies.

ALT, alanine transaminase; AUC, area under the curve; CRP, C-reactive protein; CT, computed tomography; ICH intracerebral hemorrhage; ICU, intensive care unit; ITECP, telerehabilitation platform; ML, machine learning; QoL, quality of life; RCT randomized controlled trial; RF random forest; HICH, hypertensive intracerebral hemorrhage; FUNC, functional outcome.

### AI applications across the phases of ICH care

#### AI applications in the acute phase: prognosis and mortality prediction during hospitalization

Across studies conducted in acute care settings, AI models, particularly those based on machine learning algorithms such as Random Forest (RF), XGBoost, and logistic regression, have demonstrated superior performance in predicting short- and long-term outcomes in patients with ICH compared to conventional scoring systems. For example, Nie et al. (14) developed machine learning models, including RF and AdaBoost, to predict in-hospital mortality using physiological data collected during the first 24 h of intensive-care unit admission. The RF model achieved an area under the curve (AUC) of 0.819, significantly outperforming the APACHE II scoring system (AUC, 0.423) in predicting in-hospital mortality. This suggests that ML-based models may offer more accurate early risk stratification than traditional tools in acute clinical settings.

Similarly, Xu et al. (17) extracted radiomic features from computed tomography (CT) images obtained at admission and applied RF and XGBoost models to predict six-month functional outcomes in patients with hypertensive ICH. The RF model achieved a sensitivity of 93.3%, a specificity of 92.5%, and an overall accuracy of 92.7%. These metrics indicate a strong predictive performance for functional outcome classification based on initial imaging data. Zhou et al. (19) combined radiomics and clinical variables to construct a logistic regression-based nomogram for predicting 30-day outcomes in patients with deep ICH. The model was validated internally and externally, yielding AUCs of 0.80 (development), 0.79 (internal validation), and 0.70 (external validation). This level of validation supports the potential generalizability of their nomogram across different patient cohorts. The models reviewed in the acute phase were primarily designed to support early prognosis and mortality prediction using physiological or imaging data acquired during initial hospitalization.

#### AI applications in the post-acute phase: post-discharge support, rehabilitation, and data completion

In the post-acute phase, AI tools have been utilized for applications such as discharge data completion, rehabilitation support, and long-term outcome prediction, reflecting a shift toward the continuity of care and patient monitoring. Studies focusing on the post-acute phase have explored the use of AI tools to enhance the continuity of care, facilitate rehabilitation, and address data limitations in patient records. Wang et al. (16) addressed the challenge of incomplete discharge assessments using ML-based imputation models and ensemble learning-based imputation techniques to restore missing discharge evaluation data. This approach demonstrated an AUC of 0.924 and sensitivity of 0.908, indicating high reliability in reconstructing incomplete clinical datasets. These results highlight the practical utility of AI for improving data quality in post-discharge care environments.

In terms of rehabilitation support, Zhang et al. (18) developed and implemented a WeChat-based telerehabilitation platform (ITECP) for young ICH survivors. Although the outcome data are not yet available, the intervention aimed to improve adherence to physical rehabilitation and enhance functional recovery and quality of life after discharge. As the RCT is still ongoing, the effectiveness of this intervention is yet to be determined, but it represents a promising direction for remote, technology-assisted rehabilitation.

Additionally, Sonobe et al. (15) implemented a Balanced RF model to predict functional outcomes after inpatient rehabilitation. Using clinical and imaging data, the model classified outcomes into three categories with AUCs of 0.952 (good), 0.790 (moderate), and 0.921 (poor), demonstrating a high predictive performance in discharge-level outcome prediction. This stratification may assist clinicians in tailoring rehabilitation plans based on individual outcome probabilities.

Together, these studies highlight the potential of AI applications beyond hospitalization, not only in augmenting clinical documentation but also in supporting patient-centered, technology-driven rehabilitation strategies. Katsuki et al. (6) employed a commercially available deep learning tool (Prediction One) to predict the 6-month functional outcomes after hypertensive ICH surgery. Their model achieved high accuracy (AUC = 0.997 training; 0.884 validation), outperforming conventional prognostic scores such as ICH and FUNC scores. Katsuki's study demonstrated that commercially available AI platforms can achieve high predictive accuracy, suggesting their potential usability in clinical settings without requiring custom model development.

Across all studies, machine learning models consistently outperformed conventional scoring systems and offered enhanced predictive capabilities using early clinical or imaging data. Radiomics-based approaches have demonstrated a particularly high accuracy. Moreover, AI-based imputation (16) effectively addresses data completeness issues, and the implementation of AI in rehabilitation (15, 18) indicates promising directions for post-ICH care.

Although none of the studies directly focused on AI-based nursing interventions, the findings suggest that AI tools can indirectly support nursing decisions by improving prognostic assessment, documentation accuracy, and post discharge planning for management of patients with ICH.

## Discussion

This narrative literature review aimed to explore the current applications of artificial intelligence (AI) in the clinical and nursing management of patients with intracerebral hemorrhage (ICH). The integration of AI into the management of ICH has shown promising advancements, particularly in prognostic assessment and post-discharge rehabilitation planning. Given the high risk of mortality and functional disability, effective prognostication and prevention of recurrence after discharge are critical components of ICH management (20, 21). In particular, ICH recurrence is a major determinant of long-term prognosis, and early identification and intervention of modifiable risk factors can improve the outcomes of ICH survivors (20).

Studies have demonstrated that machine learning models such as RF and XGBoost outperform traditional scoring systems in predicting patient outcomes during the acute phase of ICH. For instance, Nie et al. (14) developed an RF model using physiological data from the first 24 h of ICU admission, achieving an AUC of 0.819, significantly surpassing the APACHE II score’s AUC of 0.423. Similarly, Xu et al. (17) utilized radiomic features from admission CT images, with their RF model attaining a sensitivity of 93.3%, specificity of 92.5%, and overall accuracy of 92.7% for six-month functional outcome predictions. These findings underscore the enhanced predictive capabilities of AI models in acute-care settings.

In the post-acute phase, AI applications have focused on addressing data completeness and supporting rehabilitation efforts. Wang et al.16 employed ensemble learning-based imputation techniques to restore missing discharge evaluation data, demonstrating strong reliability in reconstructing incomplete clinical datasets (AUC = 0.924; sensitivity = 0.908). Zhang et al. (18) developed a WeChat-based telerehabilitation platform aimed at improving adherence to physical rehabilitation among young ICH survivors. While the platform has been implemented, the study is still in progress, and final results have not yet been peer-reviewed or published. One of the included studies was a randomized controlled trial protocol, which was selected because it represented a novel application of AI-supported telerehabilitation in ICH care—a topic scarcely addressed in the existing literature. While outcome data were not yet available, the protocol was included to reflect emerging directions in post-acute management and to highlight the potential role of mobile-based platforms. Nonetheless, the absence of peer-reviewed results limits its evaluative contribution, and this should be interpreted with caution.

Despite these advancements, our review identified a notable gap in the literature concerning AI applications directly tailored to nursing practices for the care of patients with ICH. Although current AI models primarily focus on diagnostic and prognostic assessments, their integration into nursing workflows remains limited. However, the indirect benefits of AI in nursing care should not be ignored. Enhanced prognostic assessments can inform nursing care plans, allowing for personalized and timely interventions. Improved documentation accuracy through AI-driven data completion can reduce the administrative burden on nursing staff, enabling them to allocate more time to patient care (22). Furthermore, AI-supported post-discharge planning can facilitate smoother transitions from hospital to home care, ensuring that nursing interventions align with predicted patient needs and potential complications (23).

Broader literature supports the potential of AI in transforming stroke care. A systematic review by Yang et al. (24) assessed the accuracy of AI models in predicting stroke outcomes and concluded that AI could serve as a valuable tool for clinicians in prognostic evaluation. Additionally, studies have highlighted the role of AI in stroke rehabilitation, emphasizing its capacity to personalize treatment plans and effectively monitor patient progress. For example, a review by Rahman et al. (25) discussed the application of AI in stroke rehabilitation, noting its potential to enhance patient outcomes through tailoring. These findings align with the present review in suggesting that AI may serve as a valuable adjunct to interdisciplinary stroke care.

## Conclusions and limitations

Several limitations of the included studies should be acknowledged. Most used retrospective designs with limited external validation, reducing the generalizability of their findings. For example, Katsuki et al. (6) applied a proprietary deep learning model without disclosing its structure, making it difficult to assess its interpretability or practical applicability. Similarly, Wang et al.'s (16) imputation model relied on simulated missing data, which may not fully capture the complexity of real-world clinical settings.

Furthermore, all of the included studies were conducted in China or Japan, which may limit the applicability of the findings to other healthcare systems with different infrastructures, clinical practices, or patient demographics. Future research should aim to include studies from a broader range of countries to better capture the global relevance of AI tools in ICH care.

Although a comprehensive search strategy was employed, only two studies were identified through database searches. This may reflect limitations in current indexing practices, as relevant AI studies in ICH care are often embedded within broader stroke research or described using heterogeneous terminology. It is also possible that some relevant studies were not retrieved due to variability in keyword usage across disciplines. To address this, a manual search was conducted to supplement the database results, but future reviews may benefit from expanded search terms and database-specific thesaurus mapping to improve retrieval sensitivity.

In addition, the rationale for model selection was not always clearly stated. Although algorithms such as RF and XGBoost showed high accuracy, few studies discussed their clinical usability or explainability—factors that are critical for real-world implementation. Moreover, most studies reported model performance primarily using AUC scores, without providing other clinically meaningful metrics such as sensitivity, specificity, precision, recall, or calibration. While AUC is a useful measure of overall model discrimination, it does not capture how well a model performs at specific decision thresholds or in clinically relevant subgroups. Future studies should incorporate a more comprehensive set of performance indicators to better inform practical deployment in diverse healthcare settings.

Importantly, none of the included studies directly addressed nursing-specific applications of AI. Current AI tools are primarily focused on diagnosis and prognosis, with limited attention to care coordination, patient education, or psychosocial support—areas that are central to nursing practice. Integrating nursing perspectives into the development of AI tools will be essential to ensure their relevance and effectiveness within interdisciplinary care teams.

Despite the limitations noted above, this narrative literature review highlights the emerging role of AI in enhancing the clinical and nursing management of patients with ICH across different phases of care. Although AI-based nursing interventions were not directly addressed in the included studies, the findings underscore the indirect yet significant potential of AI to inform nursing practice—through better prognostication, improved documentation, and individualized care planning. As evidence continues to accumulate, future research should explore the integration of AI tools into nursing workflows and evaluate their impact on patient-centered outcomes in ICH care.

## Data Availability

The original contributions presented in the study are included in the article/Supplementary Material, further inquiries can be directed to the corresponding author.
