# Supplementary material for: Artificial intelligence applications in intracerebral hemorrhage care: implications for clinical and nursing practice - a narrative literature review
Source: Front Rehabil Sci. 2025 Jul 7;6:1620335. doi: 10.3389/fresc.2025.1620335 (PMC12277309; doi:10.3389/fresc.2025.1620335)
Supplement: Supplementary file 1 [file Datasheet1.docx]

## Supplementary File 1: Search strategy for the included databases

**Database: PubMed (n=10)**

| **No.** | **Search Strategy** | **Results** |
| --- | --- | --- |
| #1 | ("Intracranial Hemorrhage" OR "ICH" OR "Cerebral Hemorrhage" OR "Brain Hemorrhage" OR "Traumatic Intracranial Hemorrhage" OR "Traumatic Brain Hemorrhage") OR ("Hemorrhagic Stroke"[MeSH] OR "Brain Injuries"[MeSH] OR "Craniocerebral Trauma"[MeSH]) | 253,852 |
| #2 | ("Nursing Care"[MeSH] OR "Patient-Centered Care"[MeSH] OR "Nurse-Led Care" OR "Primary Health Care"[MeSH] OR "Community Health Nursing"[MeSH] OR "Rehabilitation Nursing" OR "Nursing interventions") | 364,703 |
| #3 | ("Artificial Intelligence"[MeSH] OR "Machine Learning" OR "AI in Healthcare" OR "Predictive Analytics" OR "Deep Learning" OR "Clinical Decision Support") OR ("Digital Technology"[MeSH] OR "Telemedicine"[MeSH] OR "Remote Monitoring" OR "Digital Health" OR "E-Health" OR "Wearable Devices") | 440,320 |
| #4 | (#1 AND #2 AND #3) | 49 |
| #5 | #4 (Limiters - Published Date: 2014.01.01-2024.12.31; English Language; Free Full Text) | 10 |

**Database: CINAHL Plus with Full Text (n= 55)**

| **No.** | **Search Strategy** | **Results** |
| --- | --- | --- |
| S1 | ("Intracranial Hemorrhage" OR "Cerebral Hemorrhage" OR "Brain Hemorrhage" OR "ICH" OR "Intracranial Bleeding" OR "Traumatic Intracranial Hemorrhage" OR "Traumatic Brain Hemorrhage" OR "Hemorrhagic Stroke" OR "Stroke") | 160,429 |
| S2 | ("Nursing Care" OR "Patient-Centered Care" OR "Primary Health Care" OR "Community Health Nursing" OR "Rehabilitation Nursing" OR "Nurse-Led Care" OR "Nursing Practice" OR "Nursing Interventions") | 303,322 |
| S3 | ("Artificial Intelligence" OR "Machine Learning" OR "Deep Learning" OR "Predictive Analytics" OR "AI in Healthcare" OR "Clinical Decision Support") OR ("Digital Technology" OR "Telemedicine" OR "Remote Monitoring" OR "E-Health" OR "Digital Health" OR "Wearable Devices" OR "Health Information Technology") | 99,065 |
| S4 | (S1 AND S2 AND S3) | 79 |
| S5 | S4 (Limiters - Published Date: 2014.01.01-2024.12.31; English Language; Full Text) | 55 |

**Database: Ovid MEDLINE (n= 117)**

| **No.** | **Search Strategy** | **Results** |
| --- | --- | --- |
| S1 | (exp Intracranial Hemorrhages/ OR exp Brain Hemorrhage/ OR "Cerebral Hemorrhage".ab,ti. OR "Intracranial Bleeding".ab,ti. OR "Acute Brain Hemorrhage".ab,ti. OR "ICH".ab,ti.) | 94,007 |
| S2 | (exp Artificial Intelligence/ OR exp Machine Learning/ OR "Deep Learning".ab,ti. OR "AI in healthcare".ab,ti. OR "Predictive Analytics".ab,ti.) OR (exp Digital Technology/ OR exp Telemedicine/ OR "Remote Monitoring".ab,ti. OR "Digital Health Innovation".ab,ti. OR "Wearable Devices".ab,ti. OR "E-health".ab,ti.) | 290,991 |
| S3 | (exp Nursing Care/ OR exp Patient-Centered Care/ OR exp Rehabilitation Nursing/ OR "Nurse-Led Care".ab,ti. OR "Nursing Interventions".ab,ti. OR "Personalized Nursing Care".ab,ti.) | 172,862 |
| S4 | (S1 AND S2 AND S3 AND S4) | 1,126 |
| S5 | (S1 AND S2) AND S3 (Limiters - Free Full Text; Published Date: 2014 – 2024; Language: English) | 117 |

**Database: ProQuest Central (n=111)**

| **No.** | **Search Strategy** | **Results** |
| --- | --- | --- |
| S1 | ("Intracranial Hemorrhage" OR "ICH" OR "Cerebral Hemorrhage" OR "Traumatic Brain Hemorrhage") | 1,447,613 |
| S2 | ("Nursing Care" OR "Patient-Centered Care" OR "Community Health Nursing" OR "Nurse-Led Care") | 557,610 |
| S3 | ("Artificial Intelligence" OR "Digital Technology" OR "Remote Monitoring") | 4,663,686 |
| S4 | (S1 AND S2 AND S3) | 394 |
| S5 | S4 (Limiters - Full Text; Peer reviewed; Published Date: 2014.01.01 – 2024.12.31; Language: English) | 111 |

**Database: Web of Science (n=11)**

| **No.** | **Search Strategy** | **Results** |
| --- | --- | --- |
| S1 | TS=("Intracranial Hemorrhage" OR "ICH" OR "Cerebral Hemorrhage" OR "Brain Hemorrhage" OR "Intracranial Bleeding" OR "Acute Brain Hemorrhage" OR "Traumatic Intracranial Hemorrhage" OR "Traumatic Brain Hemorrhage" OR "Traumatic Brain Injury" OR "Hemorrhagic Stroke" OR "Stroke" OR "Acute Stroke" OR "Brain Stroke") | 347,709 |
| S2 | TS=("Nursing Care" OR "Patient-Centered Care" OR "Rehabilitation Nursing" OR "Community Health Nursing" OR "Primary Health Care" OR "Nurse-Led Care" OR "Nursing Interventions" OR "Personalized Nursing Care" OR "Post-Stroke Nursing") | 44,680 |
| S3 | TS=("Artificial Intelligence" OR "Machine Learning" OR "Deep Learning" OR "Predictive Analytics" OR "AI in Healthcare" OR "Clinical Decision Support" OR "Digital Technology" OR "Telemedicine" OR "Remote Monitoring" OR "Digital Health" OR "E-Health" OR "Wearable Devices" OR "Digital Health Innovation") | 950,672 |
| S4 | (S1 AND S2 AND S3) | 22 |
| S5 | S4 (Limiters - Full Text; Peer Reviewed; Published Date: 2014.01.01 – 2024.12.31; Language: English) | 11 |
