# Supplementary material for: Artificial intelligence applications in intracerebral hemorrhage care: implications for clinical and nursing practice - a narrative literature review
Source: Front Rehabil Sci. 2025 Jul 7;6:1620335. doi: 10.3389/fresc.2025.1620335 (PMC12277309; doi:10.3389/fresc.2025.1620335)
Supplement: Supplementary file 2 [file Datasheet2.docx]

## Supplementary File 2: List of excluded full-text studies and the reasons for exclusion

**Full-text studies and the reasons for exclusions (n=44)**

| **Excluded full-text studies (n=44)** | **Reasons for Exclusion** |
| --- | --- |
| Anderson et al., 2013 | Irrelevant study topic  (The study focuses on a web-based clinical decision support tool for preventing ischemic stroke or TIA through self-management, with an emphasis on stroke prevention decision-making. There is a lack of mention or specific use of AI technology or related technologies, making it irrelevant to the research topic.) |
| Bagot et al., 2018 | Irrelevant study topic  (The study primarily focuses on the sustainability of an acute stroke telemedicine program, which is centered on telehealth technology for stroke management, making it irrelevant to the research topic.) |
| Bayona et al., 2020 | Irrelevant study topic  (The study focuses on the development of a tele-stroke network algorithm for optimizing healthcare delivery in cerebrovascular diseases, with an emphasis on clinical pathways, patient transport, and reperfusion treatment decisions, making it irrelevant to the research topic.) |
| Brands et al., 2022 | Irrelevant study topic  (The study focuses on the impact of patient-centered digital health records on health outcomes, specifically examining the effects of PHR on self-management and treatment adherence. While it could be relevant to self-management in certain conditions, it is not directly related to patients with intracerebral hemorrhage, making it irrelevant to the research topic.) |
| Chau et al., 2019 | Irrelevant study topic  (The study focuses on the effectiveness and cost-effectiveness of a virtual multidisciplinary stroke clinic for stroke survivors and their caregivers, primarily emphasizing rehabilitation, self-management support, and community-based care services. This does not directly align with the research topic on intracerebral hemorrhage.) |
| Chen et al., 2014 | Irrelevant study topic  (This study evaluates a teleconsultation system using store-and-forward technology for wound assessment. Although it demonstrates feasibility in remote care, the intervention does not involve any artificial intelligence or machine learning techniques as defined by the review’s inclusion criteria.) |
| Chen et al., 2023 | Irrelevant study topic  (The study focuses on predicting rehabilitation outcomes for ischemic and hemorrhagic stroke patients, with an emphasis on building prediction models using machine learning algorithms. It is centered on data analysis and model performance evaluation, making it irrelevant to the research topic on intracerebral hemorrhage.) |
| Coote et al., 2022 | Irrelevant study topic  (The study investigates the expanded scope of practice for nurses in mobile stroke units (MSUs), focusing on staffing models and role development. However, the study does not involve artificial intelligence or AI-based technologies as defined in the review's inclusion criteria.) |
| Dave et al., 2018 | Irrelevant study topic  (The study focuses on the costs and outcomes of stroke within the military healthcare system, presenting the need for a stroke model through cost and outcome analysis. However, AI technology is not utilized, making it irrelevant to the research topic on intracerebral hemorrhage.) |
| Davoody et al., 2016 | Irrelevant study topic  (The study focuses on the information needs of stroke patients post-discharge, with an emphasis on mild and rehabilitative stroke patients. There is no clear mention of hemorrhagic stroke being included in the study, making it irrelevant to the research topic on intracerebral hemorrhage.) |
| dos Santos et al., 2024 | Irrelevant study topic  (The study is an overview of systematic reviews evaluating tele-interventions for behavior change and self-management in secondary stroke prevention. While it shows positive outcomes, no artificial intelligence technologies were used or assessed, and therefore the study does not meet the inclusion criteria focused on AI-based applications in ICH care.) |
| Erkelens et al., 2020 | Irrelevant study topic  (The study primarily evaluates the accuracy of the Netherlands Triage Standard (NTS), a decision support tool, for transient ischemic attacks and stroke. It does not focus on AI technology itself or provide specific examples of AI integration in the nursing process, making it irrelevant to the research topic.) |
| Fernandez-Lozano et al., 2021 | Irrelevant study topic  (The study develops a predictive model using logistic regression and support vector machine based on clinical and CT data to estimate ICH patient outcomes. However, the model is not applied in a clinical workflow or nursing context, and therefore does not meet the inclusion criteria.) |
| Flanders, 2019 | Irrelevant study topic  (The study primarily focuses on ischemic stroke patients, examining the application of a tele-stroke program and process improvements in the emergency department (ED). It lacks specific discussion on the role of nurses or the application of digital transformation tools in nursing management, making it irrelevant to the research topic on intracerebral hemorrhage.) |
| Fowler et al., 2019 | Irrelevant study topic  (The study focuses on acute ischemic stroke and examines the use of a nurse-led telehealth team. While a telemedicine system is used, there is no clear implementation of AI technology, making it irrelevant to the research topic on intracerebral hemorrhage.) |
| Hassan et al., 2022 | Irrelevant study topic  (The study focuses on reducing stroke transfer times in primary care settings using AI, targeting stroke patients rather than intracerebral hemorrhage patients. It is more focused on healthcare collaboration and improving patient care pathways rather than the relationship between AI and nursing task optimization, making it irrelevant to the research topic.) |
| He et al., 2020 | Irrelevant study topic  (The study develops a predictive model using logistic regression and support vector machine based on clinical and CT data to estimate ICH patient outcomes. However, the model is not applied in a clinical workflow or nursing context, and therefore does not meet the inclusion criteria.) |
| Kleinpell et al., 2015 | Irrelevant study topic  (The study focuses on the impact of telemedicine on ICU nursing, with an emphasis on changes in nurse tasks in the ICU. It does not address the relevance of intracerebral hemorrhage patients or AI, making it irrelevant to the research topic.) |
| Kritz, 2019 | Irrelevant study topic  (The study addresses the impact of digital transformation on nursing tasks and patient care but does not explicitly include intracerebral hemorrhage patient management, making it irrelevant to the research topic.) |
| Lyerly et al., 2021 | Irrelevant study topic  (The study focuses on provider communication and the impact of tele-stroke consultations, with stroke patients as the overall study population. The ratio of ICH and IS patients is not clearly distinguished, and the study focuses more on telemedicine rather than the role of digital technology, making it irrelevant to the research topic.) |
| Michalski et al., 2021 | Irrelevant study topic  (The study evaluates the feasibility of a post-stroke management platform that uses digital devices and telemonitoring. However, it does not employ artificial intelligence, machine learning, or predictive modeling technologies as defined in the inclusion criteria, and thus does not align with the research focus on AI-based applications in ICH care.) |
| Moreira et al., 2021 | Irrelevant study topic  (The study focuses on automating stroke patient transfer pathways, with only 13.4% of patients being intracerebral hemorrhage cases. The emphasis is on hospital intra-movement and the measurement of patient care time by healthcare providers, making it more focused on hospital operations and pathway optimization rather than digital nursing application, thus irrelevant to the research topic.) |
| Nawabi et al., 2021 | Irrelevant study topic  (This study uses a machine learning model based on CT-derived imaging features to predict functional outcomes in ICH patients. Although AI technology is employed, the study focuses solely on model performance and lacks clinical or nursing application, thus not meeting the inclusion criteria.) |
| Olson et al., 2022 | Irrelevant study topic  (The study primarily focuses on the management of ischemic stroke and evaluates a nurse-led tele-stroke protocol in the emergency department, making it irrelevant to the research topic on intracerebral hemorrhage.) |
| Peng et al., 2010 | Irrelevant study topic and publication year outside the inclusion range  (The study develops machine learning models for predicting 30-day mortality in SICH patients but does not demonstrate clinical application. Moreover, it was published in 2010, which is outside the defined inclusion period of 2014–2024.) |
| Purvis et al., 2022 | Irrelevant study topic  (The study provides a design case for digital technology application in self-management support for stroke survivors, which could be referenced. However, it lacks direct relevance to intracerebral hemorrhage patients and focuses on self-management and psychological support, with limited research on nursing efficiency.) |
| Rai et al., 2020 | Irrelevant study topic  (The study focuses on digital interventions for blood pressure self-management and medication adjustment in stroke patients, with some intracerebral hemorrhage patients included. However, the emphasis is on blood pressure management rather than nursing management, making it irrelevant to the research topic.) |
| Ramaswamy et al., 2023 | Irrelevant study topic  (The study focuses on a mobile application for stroke survivors, with an emphasis on stroke survivors themselves. It does not address enhancing the role of nurses through AI or improvements in nursing care through AI application, making it irrelevant to the research topic.) |
| Ranta et al., 2015 | Irrelevant study topic  (The study focuses on electronic decision support for transient ischemic attack (TIA) in primary care settings. It is unrelated to nursing management of intracerebral hemorrhage patients and primarily evaluates the effectiveness of electronic decision support tools for general practitioners.) |
| Ranta et al., 2014 | Irrelevant study topic  (The study focuses on an electronic decision support tool for transient ischemic attack (TIA) in primary care settings. While the AI-based system provides useful data on patient care processes, it targets TIA and mild ischemic stroke patients, making it irrelevant to the research topic on intracerebral hemorrhage.) |
| Richardson et al., 2016 | Irrelevant study topic  (The study focuses on evaluating the completeness of nursing documentation for stroke patients in the emergency department, with an emphasis on ischemic and hemorrhagic stroke. It does not directly address intracerebral hemorrhage nursing management, making it irrelevant to the research topic.) |
| Rosenberg, 2016 | Irrelevant study topic  (The study primarily focuses on the role of nursing and its effectiveness, as well as the barriers to telemedicine in critical care environments. It does not specifically address nursing management for intracerebral hemorrhage patients or nursing care through digital transformation, making it irrelevant to the research topic.) |
| Sakakibara et al., 2017 | Irrelevant study topic  (The study focuses on the development of a chronic disease management program for stroke survivors, not specifically targeting intracerebral hemorrhage patients. It is focused on developing a program for stroke patients in general, making it irrelevant to the research topic.) |
| Schuelke et al., 2020 | Irrelevant study topic  (The study evaluated the effects of a virtual nursing team on missed care using a human-centered remote monitoring approach. However, no AI-based technologies—as defined by machine learning, predictive modeling, or automated decision-support—were applied. Thus, the study does not meet the inclusion criteria.) |
| Tan et al., 2024 | Irrelevant study topic  (The study focuses on digital health stroke management in primary care settings, but it does not target intracerebral hemorrhage patients. Only 13.5% of the participants have hemorrhagic stroke, with the main focus on ischemic stroke, making it irrelevant to the research topic.) |
| Teng et al., 2021 | Irrelevant study topic  (The study focuses on developing an artificial intelligence model for predicting early hematoma expansion in intracerebral hemorrhage patients. However, it does not directly align with the research topic of nursing management or the use of digital transformation in nursing for these patients.) |
| Vilendrer et al., 2022 | Irrelevant study topic  (The study focuses on changes in nursing workflow following the introduction of telemedicine in a COVID-19 ward, which is a specific context where nurses use digital technology to modify workflows. While meaningful, it lacks direct relevance to intracerebral hemorrhage patients.) |
| Wang et al., 2019 | Irrelevant study topic  (The study focuses on developing a machine learning-based prognostic model for functional outcomes in ICH patients using AutoML and random forest. However, it does not involve clinical application, nursing intervention, or integration into patient care, and therefore does not meet the review’s inclusion criteria.) |
| Weber et al., 2020 | Irrelevant study topic  (The study focuses on the development of a telemedicine network and emergency care improvement for stroke and other neurological emergencies in northeastern Germany. It does not include nursing management for intracerebral hemorrhage patients or the application of digital transformation tools in nursing care, making it irrelevant to the research topic.) |
| Williams et al., 2019 | Irrelevant study topic  (The study focuses on preventing crises through nursing interventions in a tele-ICU environment, with an emphasis on tele-ICU care and intervention. It does not specifically address intracerebral hemorrhage patients or the application of digital transformation technologies in nursing care, making it irrelevant to the research topic.) |
| Wu et al., 2020 | Irrelevant study topic  (The study focuses on evaluating the effectiveness of a remote rehabilitation exercise program for acute stroke patients, with an emphasis on rehabilitation and functional recovery. It does not specifically address intracerebral hemorrhage patients or nursing management, making it irrelevant to the research topic.) |
| Yan et al., 2021 | Irrelevant study topic  (The study focuses on mobile health interventions for stroke management in rural China, with 13.6% of participants being intracerebral hemorrhage patients. However, the intervention is primarily led by village doctors, with no discussion on nurse-led management or digital transformation in nursing care. The focus is mainly on supporting primary care through mHealth apps, making it irrelevant to the research topic.) |
| Yang et al., 2021 | Irrelevant study topic  (The study focuses more on acute stroke patients, primarily ischemic, rather than intracerebral hemorrhage patients, and lacks specific connections to nursing practice through digital technology, making it irrelevant to the research topic.) |
| Yang et al., 2023 | Irrelevant study topic  (The study explores the use of a rule-based nursing information system to analyze nursing diagnosis patterns in cerebral hemorrhage patients. However, rule-based systems are not considered artificial intelligence under the defined inclusion criteria. Therefore, the study does not meet the eligibility requirements focused on AI-based technologies in ICH care.) |

**References of excluded full-text studies**

Anderson, J. A., Godwin, K. M., Saleem, J. J., Russell, S., Robinson, J. J., & Kimmel, B. (2013). Accessibility, usability, and usefulness of a web-based clinical decision support tool to enhance provider–patient communication around self-management to prevent (stop) stroke. *Health Informatics Journal*, *20*(4), 261–274. https://doi.org/10.1177/1460458213493195

Bagot, K. L., Moloczij, N., Barclay-Moss, K., Vu, M., Bladin, C. F., & Cadilhac, D. A. (2018). Sustainable implementation of innovative, technology-based health care practices: A qualitative case study from stroke telemedicine. *Journal of Telemedicine and Telecare*, *26*(1–2), 79–91. https://doi.org/10.1177/1357633x18792380

Bayona, H., Ropero, B., Salazar, A. J., Pérez, J. C., Granja, M. F., Martínez, C. F., & Useche, J. N. (2020). Comprehensive Telestroke Network to optimize health care delivery for cerebrovascular diseases: Algorithm development. *Journal of Medical Internet Research*, *22*(7). https://doi.org/10.2196/18058

Brands, M. R., Gouw, S. C., Beestrum, M., Cronin, R. M., Fijnvandraat, K., & Badawy, S. M. (2022). Patient-centered digital health records and their effects on Health Outcomes: Systematic Review. *Journal of Medical Internet Research*, *24*(12). https://doi.org/10.2196/43086

Chau, J. P., Lo, S. H., Lee, V. W., Choi, K. C., Shum, E. W., Hung, Z. S., Mok, V. C., Siow, E. K., Ching, J. Y., Lam, S. K., Yeung, J. H., Li, S. H., & Lau, A. Y. (2019). Effectiveness and cost-effectiveness of a virtual multidisciplinary stroke care clinic for community-dwelling stroke survivors and caregivers: A randomised controlled trial protocol. *BMJ Open*, *9*(5). https://doi.org/10.1136/bmjopen-2018-026500

Chen, C.-H., Young, T.-H., Huang, C.-H., Chang, H.-H., Chen, C.-L., Chien, H.-F., Chen, J.-S., Lai, H.-S., & Cheng, N.-C. (2014). Patient-centered wound teleconsultation for cutaneous wounds. Annals of Plastic Surgery, 72(2), 220–224. https://doi.org/10.1097/sap.0000000000000031

Chen, Y.-W., Lin, K., Li, Y., & Lin, C.-J. (2023). Predicting patient-reported outcome of activities of daily living in stroke rehabilitation: A machine learning study. *Journal of NeuroEngineering and Rehabilitation*, *20*(1). https://doi.org/10.1186/s12984-023-01151-6

Coote, S., Mackey, E., Alexandrov, A. W., Cadilhac, D. A., Alexandrov, A. V., Easton, D., Zhao, H., Langenberg, F., Bivard, A., Stephenson, M., Parsons, M. W., Campbell, B. C. V., Donnan, G. A., Davis, S. M., & Middleton, S. (2022). The mobile stroke unit nurse: An international exploration of their scope of practice, education, and training. Journal of Neuroscience Nursing, 54(2), 61–67. https://doi.org/10.1097/jnn.0000000000000632

Dave, A., Cagniart, K., & Holtkamp, M. D. (2018). A case for Telestroke in military medicine: A retrospective analysis of stroke cost and outcomes in U.S. military health-care system. *Journal of Stroke and Cerebrovascular Diseases*, *27*(8), 2277–2284. https://doi.org/10.1016/j.jstrokecerebrovasdis.2018.04.018

Davoody, N., Koch, S., Krakau, I., & Hägglund, M. (2016). Post-discharge stroke patients’ information needs as input to proposing patient-centred eHealth Services. *BMC Medical Informatics and Decision Making*, *16*(1). https://doi.org/10.1186/s12911-016-0307-2

dos Santos, J. M., Peniche, P. da, Lennon, O., Hall, P., & Faria, C. D. (2024). Effectiveness of tele-interventions for behavior change and self-management in stroke secondary prevention: Overview of systematic reviews. Brazilian Journal of Physical Therapy, 28, 100791. https://doi.org/10.1016/j.bjpt.2024.100791

Erkelens, D. C., Rutten, F. H., Wouters, L. T., Dolmans, L. S., de Groot, E., Damoiseaux, R. A., & Zwart, D. L. (2020). Accuracy of telephone triage in patients suspected of transient ischaemic attack or stroke: A cross-sectional study. *BMC Family Practice*, *21*(1). https://doi.org/10.1186/s12875-020-01334-3

Fernandez-Lozano, C., Hervella, P., Mato-Abad, V., Rodríguez-Yáñez, M., Suárez-Garaboa, S., López-Dequidt, I., Estany-Gestal, A., Sobrino, T., Campos, F., Castillo, J., Rodríguez-Yáñez, S., & Iglesias-Rey, R. (2021). Random Forest-based prediction of stroke outcome. Scientific Reports, 11(1). https://doi.org/10.1038/s41598-021-89434-7

Flanders, S. (2019). Advancing telestroke interventions in an urban Ed. *Nursing*, *49*(6), 18–20. https://doi.org/10.1097/01.nurse.0000558087.69980.7e

Fowler, S. B., Rosado, C. A., Jones, J., Ashworth, S., & Adams, D. (2019). Novel use of a nurse-led telemedicine team in acute stroke: A retrospective review of the impact on a regional health care system. *Journal of Emergency Nursing*, *45*(3), 242–248. https://doi.org/10.1016/j.jen.2018.07.026

Hassan, A. E., Ringheanu, V. M., & Tekle, W. G. (2022). The implementation of artificial intelligence significantly reduces door-in-door-out times in a primary care center prior to transfer. *Interventional Neuroradiology*, *29*(6), 631–636. https://doi.org/10.1177/15910199221122848

He, X., Chen, M., Du, C., Zhao, K., Yang, M., & Ma, Q. (2020). A novel model for predicting the outcome of intracerebral hemorrhage: Based on 1186 patients. Journal of Stroke and Cerebrovascular Diseases, 29(8), 104867. https://doi.org/10.1016/j.jstrokecerebrovasdis.2020.104867

Kleinpell, R., Barden, C., Rincon, T., McCarthy, M., & Zapatochny Rufo, R. J. (2015). Assessing the impact of telemedicine on nursing care in Intensive Care Units. *American Journal of Critical Care*, *25*(1). https://doi.org/10.4037/ajcc2016808

Kritz, F. (2019). Technology is transforming work for nurses and care for patients. *AJN, American Journal of Nursing*, *119*(3), 18–19. https://doi.org/10.1097/01.naj.0000554030.43840.b3

Lyerly, M., Selch, G., Martin, H., LaPradd, M., Ofner, S., Graham, G., Anderson, J., Martini, S., & Williams, L. S. (2021). Provider communication and telepresence enhance veteran satisfaction with Telestroke consultations. *Stroke*, *52*(1), 253–259. https://doi.org/10.1161/strokeaha.120.029993

Michalski, D., Prost, A., Handel, T., Schreiber, M., Tylcz, J.-B., Geisler, D., Urban, D., Schramm, S., Lippmann, S., Gullnick, J., Neumuth, T., Classen, J., & Ivanova, G. (2021). The poststroke-manager – combining mobile, digital and sensor-based technology with personal assistance: Protocol of the feasibility study. Neurological Research and Practice, 3(1). https://doi.org/10.1186/s42466-021-00137-w

Moreira, T., Furnica, A., Daemen, E., Mazya, M. V., Sjöstrand, C., Kaijser, M., & Loenen, E. van. (2021). Staff and facility utilization in direct patient transfer to the Comprehensive Stroke Center: Testing a real-time location system for automatic patient pathway characterization. *Frontiers in Neurology*, *12*. https://doi.org/10.3389/fneur.2021.741551

Nawabi, J., Kniep, H., Elsayed, S., Friedrich, C., Sporns, P., Rusche, T., Böhmer, M., Morotti, A., Schlunk, F., Dührsen, L., Broocks, G., Schön, G., Quandt, F., Thomalla, G., Fiehler, J., & Hanning, U. (2021). Imaging-based outcome prediction of acute intracerebral hemorrhage. Translational Stroke Research, 12(6), 958–967. https://doi.org/10.1007/s12975-021-00891-8

Olson, D. M., Provencher, M., Stutzman, S. E., Hynan, L. S., Novakovic, S., Guttikonda, S., Figueroa, S., Novakovic-White, R., Yang, J. P., & Goldberg, M. P. (2022). Outcomes from a nursing-driven acute stroke care protocol for telehealth encounters. *Journal of Emergency Nursing*, *48*(4), 406–416. https://doi.org/10.1016/j.jen.2022.01.013

Peng, S. ‐Y., Chuang, Y. ‐C., Kang, T. ‐W., & Tseng, K. ‐H. (2010). Random Forest can predict 30‐day mortality of spontaneous intracerebral hemorrhage with remarkable discrimination. European Journal of Neurology, 17(7), 945–950. https://doi.org/10.1111/j.1468-1331.2010.02955.x

Purvis, T., Busingye, D., Andrew, N. E., Kilkenny, M. F., Thrift, A. G., Li, J. C., Cameron, J., Thijs, V., Hackett, M. L., Kneebone, I., Lannin, N. A., & Cadilhac, D. A. (2022). Mixed methods evaluation to explore participant experiences of a pilot randomized trial to facilitate self‐management of people living with stroke: Inspiring Virtual enabled resources following vascular events (iverve). *Health Expectations*, *25*(5), 2570–2581. https://doi.org/10.1111/hex.13584

Rai, T., Morton, K., Roman, C., Doogue, R., Rice, C., Williams, M., Schwartz, C., Velardo, C., Tarassenko, L., Yardley, L., McManus, R. J., & Hinton, L. (2020). Optimizing a digital intervention for managing blood pressure in stroke patients using a diverse sample: Integrating the person‐based approach and patient and public involvement. *Health Expectations*, *24*(2), 327–340. https://doi.org/10.1111/hex.13173

Ramaswamy, S., Gilles, N., Gruessner, A. C., Burton, D., Fraser, M. A., Weingast, S., Kunnakkat, S., Afable, A., Kaufman, D., Singer, J., Balucani, C., & Levine, S. R. (2023). User-centered mobile applications for stroke survivors (MAPPS): A mixed-methods study of patient preferences. *Archives of Physical Medicine and Rehabilitation*, *104*(10), 1573–1579. https://doi.org/10.1016/j.apmr.2023.05.009

Ranta, A., Dovey, S., Weatherall, M., O’Dea, D., Gommans, J., & Tilyard, M. (2015). Cluster randomized controlled trial of TIA electronic decision support in primary care. *Neurology*, *84*(15), 1545–1551. https://doi.org/10.1212/wnl.0000000000001472

Ranta, A., Yang, C.-F., Funnell, M., Cariga, P., Murphy-Rahal, C., & Cogger, N. (2014). Utility of a primary care based transient ischaemic attack electronic decision support tool: A prospective sequential comparison. *BMC Family Practice*, *15*(1). https://doi.org/10.1186/1471-2296-15-86

Richardson, K. J., Sengstack, P., Doucette, J. N., Hammond, W. E., Schertz, M., Thompson, J., & Johnson, C. (2016). Evaluation of nursing documentation completion of stroke patients in the emergency department. *CIN: Computers, Informatics, Nursing*, *34*(2), 62–70. https://doi.org/10.1097/cin.0000000000000206

Rosenberg, K. (2016). Telemedicine seen as a boon to critical care nursing. *AJN, American Journal of Nursing*, *116*(5), 61. https://doi.org/10.1097/01.naj.0000482970.77413.70

Sakakibara, B. M., Lear, S. A., Barr, S. I., Benavente, O., Goldsmith, C. H., Silverberg, N. D., Yao, J., & Eng, J. J. (2017). Development of a chronic disease management program for stroke survivors using intervention mapping. *Archives of Physical Medicine and Rehabilitation*, *98*(6), 1195–1202. https://doi.org/10.1016/j.apmr.2017.01.019

Schuelke, S., Aurit, S., Connot, N., & Denney, S. (2020). The effect of virtual nursing and missed nursing care. Nursing Administration Quarterly, 44(3), 280–287. https://doi.org/10.1097/naq.0000000000000419

Tan, J., Gong, E., Gallis, J. A., Sun, S., Chen, X., Turner, E. L., Luo, S., Duan, J., Li, Z., Wang, Y., Yang, B., Lu, S., Tang, S., Bettger, J. P., Oldenburg, B., Miranda, J. J., Karmacharya, B., Kinra, S., Shao, R., … Yan, L. L. (2024). Primary care–based digital health–enabled stroke management intervention. *JAMA Network Open*, *7*(12). https://doi.org/10.1001/jamanetworkopen.2024.49561

Teng, L., Ren, Q., Zhang, P., Wu, Z., Guo, W., & Ren, T. (2021). Artificial intelligence can effectively predict early hematoma expansion of intracerebral hemorrhage analyzing noncontrast computed tomography image. *Frontiers in Aging Neuroscience*, *13*. https://doi.org/10.3389/fnagi.2021.632138

Vilendrer, S., Lough, M. E., Garvert, D. W., Lambert, M. H., Lu, J. H., Patel, B., Shah, N. H., Williams, M. Y., & Kling, S. M. (2022). Nursing workflow change in a COVID-19 inpatient unit following the deployment of inpatient telehealth: Observational study using a real-time locating system. *Journal of Medical Internet Research*, *24*(6). https://doi.org/10.2196/36882

Wang, H.-L., Hsu, W.-Y., Lee, M.-H., Weng, H.-H., Chang, S.-W., Yang, J.-T., & Tsai, Y.-H. (2019). Automatic machine-learning-based outcome prediction in patients with primary intracerebral hemorrhage. Frontiers in Neurology, 10. https://doi.org/10.3389/fneur.2019.00910

Weber, J. E., Angermaier, A., Bollweg, K., Erdur, H., Ernst, S., Flöel, A., Gorski, C., Kandil, F. I., Kinze, S., Kleinsteuber, K., Kurth, T., Schmehl, I., Theen, S., Endres, M., & Audebert, H. J. (2020). Acute neurological care in north-East Germany with Telemedicine Support (annotem): Protocol of a multi-center, controlled, open-label, two-Arm Intervention Study. *BMC Health Services Research*, *20*(1). https://doi.org/10.1186/s12913-020-05576-w

Williams, L.-M. S., Nemeth, L. S., Johnson, E., Armaignac, D. L., & Magwood, G. S. (2019). Telemedicine Intensive Care Unit nursing interventions to prevent failure to rescue. *American Journal of Critical Care*, *28*(1), 64–75. https://doi.org/10.4037/ajcc2019577

Wu, Z., Xu, J., Yue, C., Li, Y., & Liang, Y. (2020). Collaborative Care Model Based Telerehabilitation Exercise Training Program for acute stroke patients in China: A randomized controlled trial. *Journal of Stroke and Cerebrovascular Diseases*, *29*(12), 105328. https://doi.org/10.1016/j.jstrokecerebrovasdis.2020.105328

Yan, L. L., Gong, E., Gu, W., Turner, E. L., Gallis, J. A., Zhou, Y., Li, Z., McCormack, K. E., Xu, L.-Q., Bettger, J. P., Tang, S., Wang, Y., & Oldenburg, B. (2021). Effectiveness of a primary care-based Integrated Mobile Health Intervention for Stroke Management in rural China (Sinema): A cluster-randomized controlled trial. *PLOS Medicine*, *18*(4). https://doi.org/10.1371/journal.pmed.1003582

Yang, R., Zhang, Y., Xu, M., & Ma, J. (2021). Image features of magnetic resonance angiography under Deep Learning in exploring the effect of comprehensive rehabilitation nursing on the neurological function recovery of patients with acute stroke. *Contrast Media &amp; Molecular Imaging*, *2021*, 1–9. https://doi.org/10.1155/2021/1197728

Yang, L., Xu, Y., Ji, X., Wang, Z., Cao, C., Chong, P., & Wu, Z. (2023). Analysis of Intensive Care Unit Nursing Clinical judgment and selection of nursing diagnosis for cerebral hemorrhage. *CIN: Computers, Informatics, Nursing*, *41*(10), 789–795. https://doi.org/10.1097/cin.0000000000001023
